# Fibronectin Promotes the Malignancy of Glioma Stem-Like Cells Via Modulation of Cell Adhesion, Differentiation, Proliferation and Chemoresistance

**DOI:** 10.3389/fnmol.2018.00130

**Published:** 2018-04-13

**Authors:** Qi Yu, Yixue Xue, Jing Liu, Zhuo Xi, Zhen Li, Yunhui Liu

**Affiliations:** ^1^Department of Neurosurgery, Shengjing Hospital of China Medical University, Shenyang, China; ^2^Liaoning Clinical Medical Research Center in Nervous System Disease, Shenyang, China; ^3^Key Laboratory of Neuro-oncology in Liaoning Province, Shenyang, China; ^4^Department of Neurobiology, College of Basic Medicine, China Medical University, Shenyang, China; ^5^Key Laboratory of Cell Biology, Ministry of Public Health of China, China Medical University, Shenyang, China; ^6^Key Laboratory of Medical Cell Biology, Ministry of Education of China, China Medical University, Shenyang, China

**Keywords:** fibronectin, glioma stem-like cells, cell adhesion-mediated drug resistance, p53, P-glycoprotein, cilengitide, focal adhesion kinase

## Abstract

Glioma stem-like cells (GSCs) are regarded as the sources of oncogenesis, recurrence, invasion and chemoresistance in malignant gliomas. Growing evidence suggests that the microenvironment surrounding GSCs interacts with tumor cells to influence biological behavior; however, the functional mechanisms involved are still unclear. In the present study, we investigated the modulation of GSCs triggered by fibronectin (FN), a main component of the extracellular matrix (ECM), in terms of cell adhesion, differentiation, proliferation and chemoresistance. We demonstrated that pre-coated FN prompted increased adherence by GSCs, with increased matrix metallopeptidases (MMPs)-2 and -9 expression, in a concentration-dependent manner. Decreases in sox-2 and nestin levels, and increased levels of glial fibrillary acidic protein (GFAP) and β-tubulin were also found in GSCs, indicating cell differentiation driven by FN. Further investigation revealed that FN promoted cell growth, as demonstrated by the elevation of Ki-67, with the activation of p-ERK1/2 and cyclin D1 also evident. In addition, FN suppressed p53-mediated apoptosis and upregulated P-glycoprotein expression, making GSCs more chemoresistant to alkylating agents such as carmustine. In contrast, this effect was reversed by an integrin inhibitor, cilengitide. Activation of the focal adhesion kinase/paxillin/AKT signaling pathway was involved in the modulation of GSCs by FN. Focusing on the interactions between tumor cells and the ECM may be an encouraging aspect of research on novel chemotherapeutic therapies in future.

## Introduction

Glioblastomas are the most common brain tumors found in humans (Jovčevska et al., 2013). With combination therapy of surgery, radiotherapy and chemotherapy (Ajaz et al., 2014), patients still exhibit a poor prognosis and outcome, with a mean survival time of only 14.6 months (Wilson et al., 2014). Increasing evidence has indicated the existence of a key population of glioblastoma cells with stem cell properties, referred to as glioma stem-like cells (GSCs; Nguyen et al., 2012), that are thought responsible for tumor genesis, the propagation of disease, the resistance to current chemotherapy and cancer recurrence (Filatova et al., 2013). Various research groups have proposed diverse hypotheses accounting for treatment failure in some patients with malignant glioma, including O^6^-methylguaninine-DNA-methytransferase gene methylation, isocitrate dehydrogenase gene mutations, aberrant ATP-binding cassette (ABC) transporter expression, p53 mutations and deletions, DNA repair deregulation, micro (mi)RNAs and long non-coding RNAs (Zeng et al., 2017). In addition to such concerns, the impact of the tumor microenvironment in various stem cell niches have been described in recent studies (Faissner and Reinhard, 2015), with several breakthroughs as a result of successfully growing stem cells on naturally-derived and synthesized substrates (Lee et al., 2012). Interactions between the extracellular matrix (ECM), adhesion molecules, soluble factors and other cells form a part of the microenvironment (Scadden, 2006). However, further investigation is required into the mechanisms involved in how the microenvironment contributes to stemness. In particular, knowledge on how the ECM regulates stemness will not only increase our understanding of regenerative medicine, but also suggest new pathways to be exploited in our efforts to counter cancer.

Fibronectin (FN) is an important ECM protein that is not only over-expressed in several cancers, but has also been shown to participate in several steps of tumorigenesis (Wang and Hielscher, 2017). Specifically, the elevation of FN has been observed in tumor samples (Caffo et al., 2004) and the peripheral blood (Sawaya et al., 1985) of glioblastoma patients. However, how FN interacts with glioma stem cells and the underlying molecular mechanisms involved are still unclear. Recently, it has been shown that miRNA-1271 inhibits cell proliferation in neuroglioma by targeting FN 1 (Gong et al., 2017), while several other studies have revealed that FN induced cell adhesion-mediated drug resistance in various kinds of tumors (Hazlehurst et al., 2006; Fei et al., 2013; Nakagawa et al., 2014), making FN a promising molecular target for chemotherapy.

In the study, we sought to explore the modulatory effect of FN on GSCs with regard to cell adhesion, differentiation, proliferation and chemoresistance, as well as their possible mechanisms.

## Materials and Methods

### Reagents and Antibodies

FN and carmustine (both from Sigma–Aldrich, St. Louis, MI, USA) stock solutions were made by dissolving these at 2 mg/mL in phosphate buffered saline (PBS) without Ca^2+^ or Mg^2+^. These were aliquoted and stored at −80°C. Basic fibroblast growth factor (b-FGF) and epidermal growth factor (EGF; both from PeproTech, Rehovot, Israel) were diluted in 0.9% NaCl. Cilengitide was purchased from Life Technologies (Carlsbad, CA, USA). For immunofluorescence staining, the following antibodies were bought: nestin (Thermo Fisher Scientific, Waltham, MA, USA), glial fibrillary acidic protein (GFAP; Aves Lab, Tigard, OR, USA), β-tubulin (Abcam, Cambridge, UK), Ki67 and sox-2 (Millipore, Billerica, MA, USA) and isotype control Ig1 (Cell Signaling Technology, Danvers, MA, USA). Primers for quantitative polymerase chain reaction (qPCR) of sox-2, β-tubulin and GAPDH were obtained from Life Technologies and primers for GFAP were from Thermo Fisher Scientific. Antibodies against GAPDH and secondary antibodies were purchased from Thermo Fisher Scientific. Primary antibodies against matrix metallopeptidase (MMP)-2/-9, t-/p-Focal adhesion kinase (FAK), p-paxillin, t-/p-AKT, P-glycoprotein, p-ERK1/2 and cyclin D1 for western blotting were purchased from Cell Signaling Technology (Danvers, MA, USA).

### Cell Lines and Culture

Glioblastoma tumor samples were collected as previously described (Yao et al., 2015). The study was carried out in accordance with the recommendations of the Ethics Committee of Shengjing Hospital of China Medical University. The protocol was approved by the Ethics Committee of Shengjing Hospital of China Medical University. All subjects gave written informed consent in accordance with the Declaration of Helsinki. Primary GBM cells were established from patients in accordance with prior work and processed as previously described (Galli et al., 2004). The human glioblastoma cell line, U87MG, was obtained from the Shanghai Institutes for Biological Sciences and Cell Resource Center, and cultured in Dulbecco’s modified Eagle’s medium (DMEM; Gibco, Thermo Fisher Scientific, Waltham, MA, USA) supplemented with 10% fetal bovine serum (FBS; Gibco). As previously reported (Yu et al., 2017), GSCs from U87MG and primary GBMs were isolated and maintained in serum-free DMEM/F12 (Gibco) containing 20 ng/mL each of EGF and bFGF, and B-27 serum-free supplement (1:50). A 5% CO_2_ humidified incubator was used to culture all cell lines at 37°C.

### Cell Adhesion Assays

GSCs were grown on FN in a Vybrant™ cell adhesion assay kit (Thermo Fisher Scientific) in accordance with the manufacturer’s instructions. FN (0 [PBS only], 1, 5 and 10 μg/mL) was coated overnight on a 96-well plate at 37°C. After pre-incubating cells with 5 μM of a fluorescent probe, calcein AM, for 30 min, wells were seeded with 10,000 cells of a calcein-labeled GSC suspension and cells incubated for another 2 h. Non-adhering cells were washed off twice with serum-free media. After adding 200 μL PBS to each well, a microplate reader was used to measure fluorescence at 490 nm (BioTek, Winooski, VT, USA). The amount of cells that adhered was measured from the fluorescence of adherent cells per well, after background fluorescence was subtracted, divided by that of added cells (after subtraction of background) multiplied by 100%.

### Cell Proliferation and Viability Assays

Cell proliferation assays were carried out using a Cell Titer 96™ tetrazolium compound (MTS) kit according to the manufacturer’s instructions. Briefly, 10,000 GSCs/well were pre-incubated in 96-well plates coated with 0, 1, 5, or 10 μg/mL FN for 24 h. Cells were then treated with 200 μM carmustine (Sigma–Aldrich, dissolved in 100% ethanol) or 1/1000 diluted ethanol (as control). Cells either formed spheres or adhered onto FN-coated wells after treatment for 72 h, after which 10 μL MTS solution was added to each well. A microplate reader (BioTek, USA) was used to measure the optical density (OD) of each well at 490 nm after incubating plates for another 2 h. Experiments were repeated three times with five replicates per experiment.

### Immunofluorescence

Cells were collected and fixed in 4% paraformaldehyde for 20 min at room temperature, rinsed in PBS twice and then in 5% BSA and 0.01% Triton X-100/PBS to permeabilize them and block protein-binding sites. The relevant primary and isotype control (as negative control) antibodies were added and cells were incubated at 4°C, overnight. Alexa-488 conjugated secondary antibodies were added at a dilution 1:500 for 2 h. Hoechst 33242 was used to stain nuclei (blue on images) and coverslips mounted on slides using mounting medium (Southern Biotech, Birmingham, AL, USA) for fluorescence. An Olympus BX61 fluorescence microscope was used to record images.

### Lentiviral Construction

Plasmid pTA-FLuc, containing a TATA-box basal promoter firefly *luciferase* reporter gene, was constructed as the normalized control, as described previously (Ariazi et al., 2007). A TATA-box promoter (TA) drove the expression of firefly *luciferase* downstream of p53-specific binding sites in multiple copies of a *cis*-acting enhancer element in a p53 reporter plasmid (Bellis et al., 2013). A p53 reporter plasmid together with lentiviral packaging vectors (pMDLGagPol, pRSV-Rev, pIVS-VSV-G) and jetPRIME (Polyplus-transfection, Illkirch, France; Duncan et al., 2014) were co-transfected into HEK-293T cells to produce lentivirus. Supernatants were collected after 48 h and centrifuged to remove cell debris. PEG-it virus concentration reagent (Systems Biosciences, Palo Alto, CA, USA) was used to concentrate the virus suspension and PBS used as a re-suspension medium.

### *Luciferase* Reporter Gene Transfection

Spinoculation procedures were used to transduce reporter vectors into cells as described previously (O’Doherty et al., 2000). Virus at a concentration of 5000 physical particles/cell was used to infect cells during centrifugation at 800× *g* for 45 min at 32°C. After removal of the supernatant, cells were resuspended in fresh medium and cultured in 24-well plates. TA–FLuc and p53-FLuc stable cell lines were created and continuously cultured for 3 days before use in a subsequent luminescence assay.

### Luminescence Assay for Transcription Factor Activity

Images of bioluminescence by firefly *luciferase* were captured by an IVIS imaging system (Caliper Life Sciences, Hopkinton, MA, USA) to evaluate transcription factor (TF) activity, as previously described (Bellis et al., 2011). After d-luciferin (1 mM; Caliper), a Fluc substrate, was added to wells, cells were incubated for 1 h. For 4 days, cells were imaged (5 min exposure) every 24 h and the medium then changed in each well. Normalized TF activity was determined by dividing the normalized light emission for p53 by the average normalized light emission for TA. Each condition was performed in triplicate.

### Apoptosis Assay by Flow Cytometry

After GSCs grown on different concentrations of FNs were treated with carmustine. A dead cell apoptosis kit (annexin V–FITC/propidium iodide (PI), Invitrogen, Carlsbad, CA, USA) was used to assay for apoptosis, according to the manufacturer’s instructions. Collected cells were washed with PBS and resuspended in 100 μL of 1× annexin-V binding buffer to 1 × 10^6^ cells/well. Annexin V–FITC (10 μL) and PI (2 μL) were added to each tube, and cells incubated in the dark for 15 min at room temperature. Analyses were performed using a BD FACS flow cytometer. Cells containing annexin V^+^/PI^−^ were defined as an early apoptotic population.

### Quantitative Real-Time PCR

An RNeasy Mini Kit (Qiagen, Hilden, Germany) was used to prepare total RNA samples following the manufacturer’s instructions. A QuantiTect^®^ SYBR Green RT–PCR Kit and a CFX384 Touch™ Real-Time PCR Detection System (Bio-Rad, Hercules, CA, USA) were used for one-step qPCR in accordance with the manufacturer’s instructions. Optical reaction plates (384-well) containing 20 ng of DNase-digested RNA per 10 μL, with 5 μL of TaqMan Universal Master mix, carboxyfluorescein (FAM)-labeled probe, and forward and reverse primers were used for reactions according to the manufacturer’s protocol. Target mRNA expression was normalized to that of GAPDH mRNA. CFX™ Manager Software 3.1 was used to generate quantification cycle (Cq) values. CFX Manager Software 3.1 was used to generate linear regression calibration curves.

### Western Blots

RIPA buffer with 0.01% of a protease and phosphatase inhibitor cocktail was used to lyse cells to prepare GSCs. A bicinchoninic acid protein assay was used to calculate protein concentrations. Sodium dodecyl sulfate–polyacrylamide gel electrophoresis on a 4%–12% gradient gel was used to separate 40 μg samples of denatured proteins. After transfer to polyvinylidene fluoride membranes, these were blocked in Pierce Protein Free blocking buffer (Thermo Fisher Scientific). Membranes were incubated overnight at 4°C with primary antibodies. Anti-GAPDH antibody was used to check for equal loading. Secondary antibodies used were horse radish peroxidase goat anti-mouse IgG or anti-rabbit IgG, and an ECL kit used to visualize immunoreactive protein bands.

### Statistical Analysis

Data were expressed as the mean ± standard deviation (SD). Graphpad Prism 6 software (San Diego, CA, USA) was used for one-way ANOVA in group comparisons to estimate statistical significance with Dunnett’s test as a *post hoc* test. Statistical significance was defined as *P* < 0.05.

## Results

### Fibronectin Promoted Cell Adhesion of Glioma Stem-Like Cells

**Figure 1:**
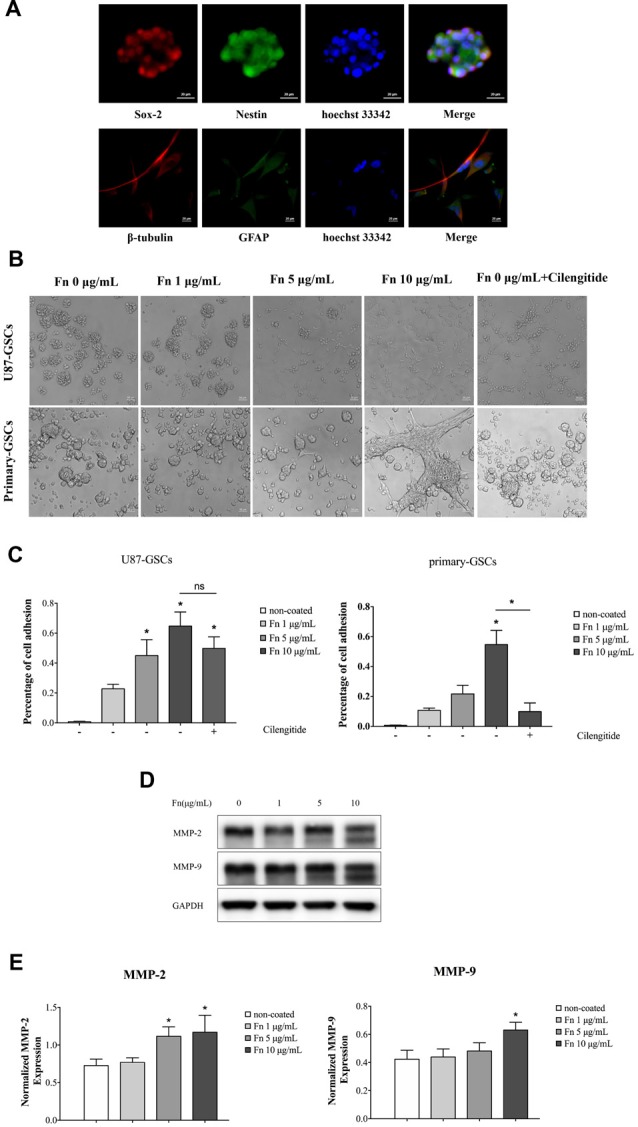
Fibronectin (FN) promoted adhesion of glioma stem-like cells (GSCs). **(A)** U87 GSCs (U87-GSCs) expressed the stemness biomarkers, sox-2 and nestin, as determined by immunofluorescence. The high expression of β-tubulin and glial fibrillary acidic protein (GFAP) was detected after GSC spheres were cultured in DMEM containing 10% fetal bovine serum (FBS). Hoechst 33242, blue nuclear stain. **(B)** U87-GSCs and primary-GSCs were cultured for 72 h on pre-coated FN that modified the growth of GSCs, in terms of their morphology and growth pattern, in a dose-dependent manner. As the concentration of FN was increased, sphere formation of GSCs decreased dramatically in serum-free medium. Cilengitide markedly detached primary-GSCs at a concentration of 100 μM.** (C)** A cell adhesion assay showed that 5 and 10 μg/mL of FN induced higher percentages of U87-GSC adhesion compared to cells not grown on FN. Only 10 μg/mL FN induced a higher percentage of primary-GSCs adhesion. The attachment of primary-GSCs, rather than U87-GSCs, was markedly reduced by cilengitide.** (D,E)** Western blots indicated higher matrix metallopeptidase (MMP)-2 and -9 expression by U87-GSCs grown on plates coated with 5 and 10 μg/mL FN. *p < 0.05, n.s. not significant.

U87-GSCs were first identified in culture by immunofluorescence staining prior to further experiments, with GSC spheres staining positively for sox-2 and nestin. Their differentiation potential was also demonstrated by the high expression of β-tubulin and GFAP after spheres were cultured in DMEM containing 10% FBS (Figure 1A). To investigate the effect of FN on cell adhesion, U87-GSC and primary-GSC spheres were trypsinized into single cells and then seeded in 96-well plates pre-coated with different concentrations of FN. After culturing for 72 h, FN modified the growth of both U87- and primary-GSCs, with regard to their morphology and growth pattern, in a dose-dependent manner. As the concentration of FN increased from 0 μg/mL to 10 μg/mL, a marked decline in sphere formation occurred, with most GSCs adhering to the bottom of the plate, even if in serum-free medium. Cilengitide, a selective integrin αV receptor antagonist (Becker et al., 2015), detached primary-GSCs rather than U87-GSCs at a concentration of 100 μM (Figure 1B). The cell adhesion assay revealed that 5 and 10 μg/mL FN significantly induced a higher percentage of U87-GSC cell adhesion compared to cells not grown on FN. Only 10 μg/mL FN induced a higher percentage of primary-GSC adhesion. The attachment of primary-GSCs, rather than U87-GSCs, was reduced significantly by cilengitide (Figure 1C). A previous study demonstrated that FN-mediated cell adhesion is required for the induction of MMP-2 and MMP-9 in human leukemia cells (Xie et al., 1998). Western blots also indicated higher MMP-2 and -9 expression by U87-GSCs in wells pre-coated with 5 and 10 μg/mL FN (Figures 1D,E).

### Fibronectin Induced Cell Differentiation of Glioma Stem-Like Cells

**Figure 2:**
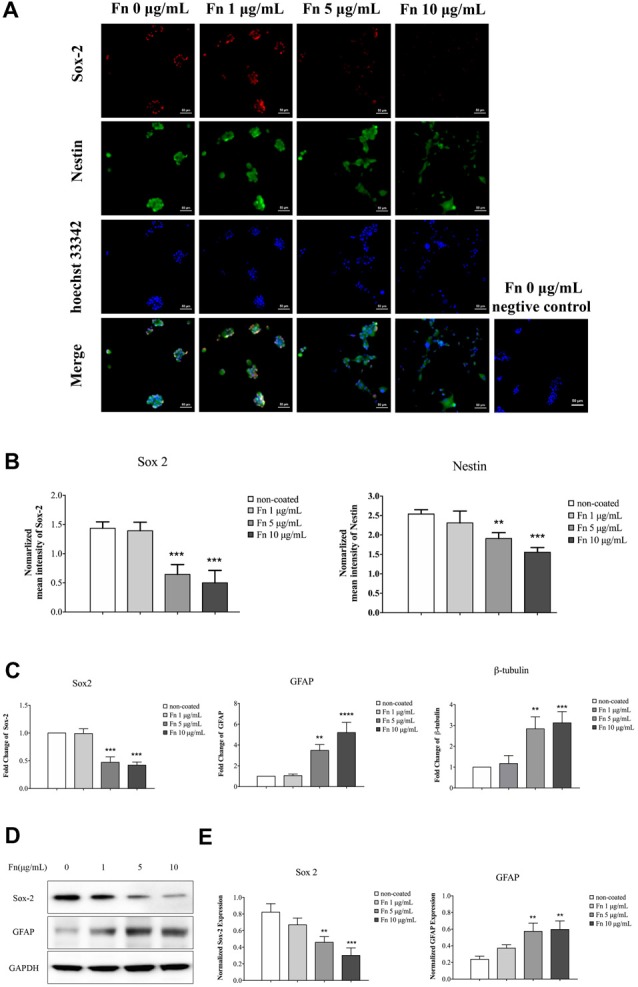
FN induced differentiation of GSCs. U87-GSCs were cultured on pre-coated FN plates (0, 1, 5 and 10 μg/mL) for 72 h. **(A,B)** Immunofluorescence staining was performed to investigate changes of the stemness markers, sox-2 and nestin. Images were taken at the same exposure settings. Cells grown without FN and stained without primary antibody were used as a negative control. Decreased expression of sox-2 and nestin was observed by cells grown on 5 and 10 μg/mL FN. Hoechst 33242, nuclear stain. **(C)** Quantitative polymerase chain reaction (qPCR) also indicated a significant decrease in sox-2 mRNA expression, and an increase of GFAP, and β-tubulin mRNA expression in cells grown on 5 and 10 μg/mL FN. **(D,E)** Furthermore, western blots confirmed significantly downregulated sox-2 and upregulated GFAP expression in U87-GSCs grown on 5 and 10 μg/mL of FN. **p < 0.01, ***p < 0.001, ****p < 0.0001.

FN has an instructive role during chondrogenesis, directing cells through the differentiation stages of cartilage formation (Singh and Schwarzbauer, 2012). As demonstrated above, FN induced morphologic changes in GSCs. To clarify whether the cell differentiation observed was accompanied by morphologic changes, immunofluorescence staining of the stemness markers, sox-2 and nestin, was performed after 72 h culture of U87-GSCs on different concentrations of FN. We found that cells showed significantly decreased expression of sox-2 and nestin when grown on 5 or 10 μg/mL FN (Figures 2A,B; *p* < 0.001 for both for sox-2; *p* < 0.01 and *p* < 0.0001 for nestin, respectively). Quantitative PCR data was in accordance with immunofluorescence data in that 5 and 10 μg/mL FN induced a significant decline in sox-2 mRNA expression (Figure 2C; *p* < 0.001 for both). In contrast, mRNA expression of GFAP and β-tubulin was significantly upregulated in cells grown on pre-coated FN plates (Figure 2C; *p* < 0.01 for both at 5 μg/mL; *p* < 0.0001 for GFAP at 10 μg/mL; *p* < 0.001 for β-tubulin at 10 μg/mL). Furthermore, western blots confirmed significantly downregulated sox-2 and upregulated GFAP expression by cells grown on FN at concentrations of 5 or 10 μg/mL, respectively (Figures 2D,E; *p* < 0.01 for both at 5 μg/mL; *p* < 0.001 for sox-2 at 10 μg/mL; *p* < 0.01 for GFAP at 10 μg/mL).

### Fibronectin Upregulated Cell Proliferation of Glioma Stem-Like Cells

**Figure 3:**
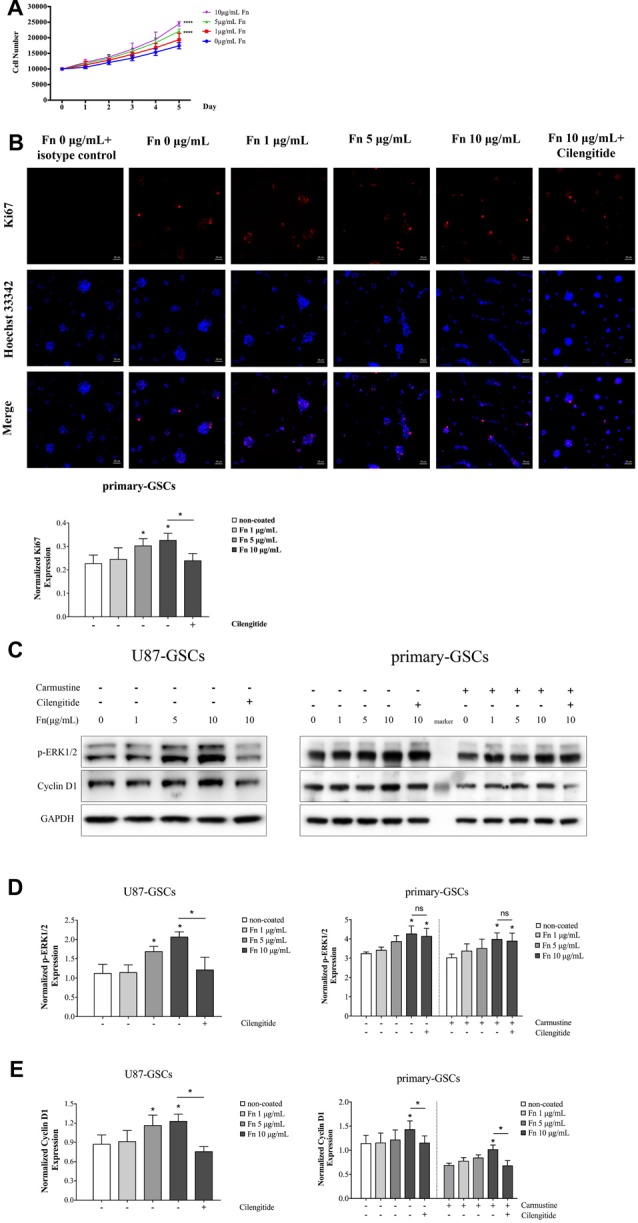
FN upregulated proliferation of GSCs. **(A)** After U87-GSCs were cultured for 72 h, cells were shown to proliferate when grown on 5 or 10 μg/mL FN. **(B)** Immunofluorescence staining revealed FN at 5 or 10 μg/mL induced increased expression of Ki-67 by primary-GSCs, indicating FN promoted cell proliferation. Whereas primary-GSCs were detached by cilengitide and Ki67 were decreased markedly comparing to that in the 10 μg/mL FN group. Images were taken at the same exposure settings. Cells grown without FN and stained with isotype control Mouse IG1 were used as a negative control. **(C–E)** Western blots showed the marked upregulation of p-ERK1/2 and cyclin D1 by U87-GSCs grown on 5 or 10 μg/mL FN. Primary-GSCs showed markedly higher expression of these two proteins when grown on 10 μg/mL FN, with or without carmustine treatment. Cilengitide significantly suppressed both *p*-ERK1/2 and cyclin D1 expression of U87-GSCs. However, cilengitide only suppressed cyclin D1 expression in primary-GSCs. *p < 0.05, n.s. not significant.

To assess the effect of FN on cell proliferation, U87-GSCs were cultured on different concentrations of FN for 72 h and proliferation curves measured using an MTS tetrazolium assay. A significant increase in U87-GSC growth was noted with cells grown on 5 or 10 μg/mL FN (Figure 3A; *p* < 0.0001 for both). Because Ki-67 protein is expressed in G_1_, S, G_2_, phases and mitosis but not resting cells (G_0_), it can be used as a marker of cell proliferation (Scholzen and Gerdes, 2000). Immunofluorescence staining of Ki-67 was performed and revealed that FN at 5 and 10 μg/mL induced significantly increased expression of Ki-67 by primary-GSCs, indicating FN promoted cell proliferation. Whereas primary-GSCs were detached by cilengitide and Ki67 were decreased markedly comparing to that in the 10 μg/mL FN group (Figure 3B; *p* < 0.05 for both). Similar results were obtained from U87-GSCs (Supplementary Figure S2). Subsequently, proteins in the proliferation-related signaling pathways, *p*-ERK1/2 and cyclin D1, were investigated using western blots. We found markedly higher expression of *p*-ERK1/2 and cyclin D1 by U87-GSCs grown on 5 or 10 μg/mL FN (Figures 3C–E; *p* < 0.05 for all). Markedly higher expression of *p*-ERK1/2 and cyclin D1 by primary-GSCs grown on 10 μg/mL FN was also observed, with or without treatment with carmustine, which prevents DNA replication and transcription (Figures 3C–E; *p* < 0.05 for all). Cilengitide significantly suppressed both *p*-ERK1/2 and cyclin D1 expression of U87-GSCs (Figures 3C–E; *p* < 0.05 for both). However, cilengitide only suppressed cyclin D1 expression of primary-GSCs (Figures 3C–E; *p* < 0.05).

### Fibronectin Inhibited p53-Mediated Apoptosis Induced by Carmustine

**Figure 4:**
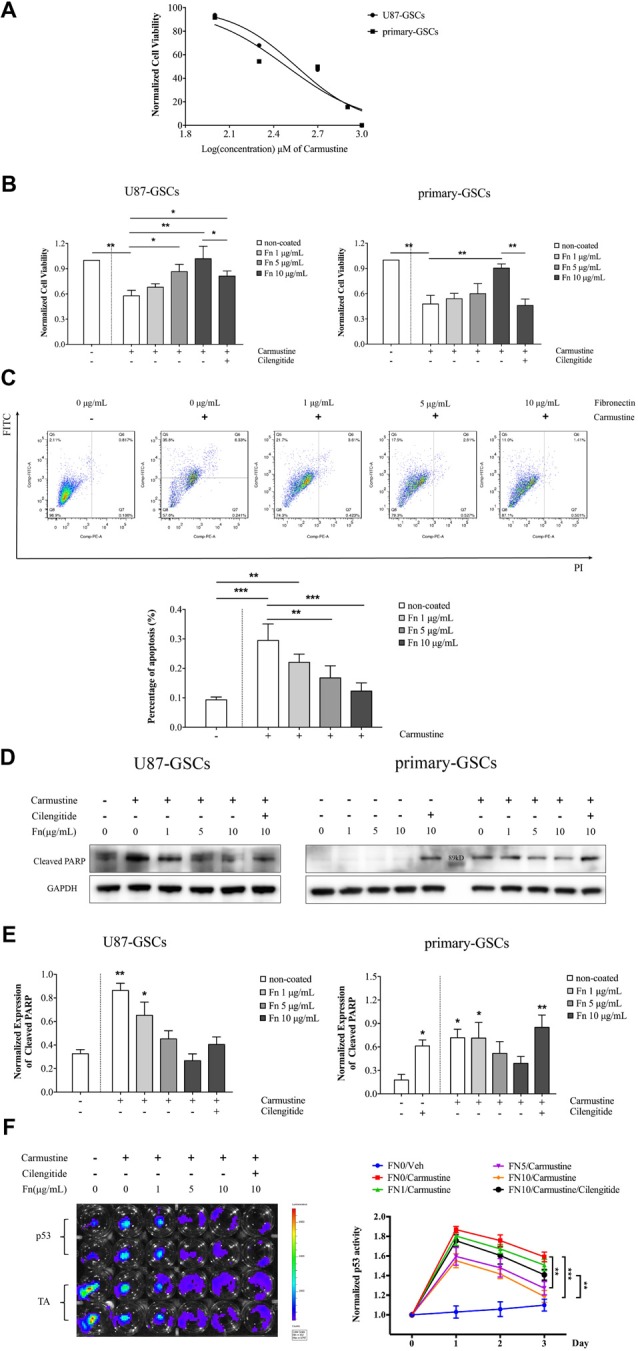
FN inhibited p53-mediated apoptosis induced by carmustine. **(A)** A dose inhibition curve of carmustine treated GSCs revealed a half maximal inhibitory concentration (IC_50_) of 504.3 ± 25.3 μM for U87-GSCs and an IC_50_ of 395 ± 19.4 μM for primary-GSCs. **(B)** Cell viability indicated that compared to the control group, U87-GSCs and primary-GSCs in the absence of FN were markedly inhibited by carmustine (*p* < 0.01 for both). Nevertheless, growing U87-GSCs on 5 or 10 μg/mL FN totally restored cell viability (*p* < 0.05 and *p* < 0.01, respectively). Only primary-GSCs grown on 10 μg/mL FN showed restored cell viability (*p* < 0.01). Compared to the cells grown on 10 μg/mL FN, cilengitide decreased cell viability significantly for both U87-GSCs and primary-GSCs (*p* < 0.05 and *p* < 0.01, respectively). **(C)** Apoptosis, as determined by flow cytometry, revealed that U87-GSCs grown on 5 or 10 μg/mL FN showed significantly decreased apoptosis induced by carmustine (*p* < 0.01, *p* < 0.001, respectively). **(D,E)** Western blots also revealed cleaved poly (ADP-ribose) polymerase (PARP) expression increased significantly in U87-GSCs grown in the absence of or on 1 μg/mL FN, but decreased in GSCs grown on 5 or 10 μg/mL FN (*p* < 0.01 and *p* < 0.05, respectively). In contrast, cilengitide reversed the anti-apoptotic effect of FN to a slight degree at a concentration of 100 μM. For primary-GSCs, cilengitide alone induced higher expression of cleaved PARP (*p* < 0.05 and *p* < 0.01 for 0 and 10 μg/mL FN, respectively). In accordance with U87-GSCs, higher levels of cleaved PARP expression were present in primary-GSCs when not grown on FN or grown on 1 μg/mL FN and treated with carmustine (*p* < 0.05 for both). In contrast to U87-GSCs, when primary-GSCs were treated with carmustine and cilengitide, they showed a markedly higher expression of cleaved PARP (*p* < 0.01). **(F)** Luciferase luminescence assays were used to determine *p53* activity. The activity of *p53* was elevated slightly without carmustine treatment in U87-GSCs grown in the absence of FN. However, *p53* activity was increased markedly by carmustine, with a peak on day 1, but declined in the following 2 days. Compared to U87-GSCs grown in the absence of FN and treated with carmustine, the *p53* activity of U87-GCSs was suppressed dramatically when grown on 5 or 10 μg/mL FN. The restoration of *p53* activity was observed when U87-GSCs were grown on 10 μg/mL FN and treated with cilengitide. *p < 0.05, **p < 0.01, ***p < 0.001.

Given that FN has been shown to induce cell adhesion–mediated drug resistance in various tumor cells (Pontiggia et al., 2012; Cho et al., 2016), we were interested in whether it had a similar capability in GSCs. The traditional chemotherapeutic reagent, temozolomide, shows very low cytotoxicity for GSCs (Yu et al., 2017). In the current study, we again used carmustine, a clinically used alkylating agent that can induced interstrand crosslinks in DNA to prevent DNA replication and transcription. First, we calculated the dose-inhibition response curve of carmustine on GSC proliferation. As shown in Figure 4A, carmustine inhibited cell viability in a dose-dependent manner, with a half maximal inhibitory concentration (IC_50_) of 504.3 ± 25.3 μM for U87-GSCs and IC_50_ of 395 ± 19.4 μM for primary-GSCs. We then treated GSCs with 200 μM carmustine to investigate whether chemoresistance was induced when cells were grown on different concentrations of FN. We found more cells survived as the concentration of FN increased. At 200 μM, increased numbers of cells died in both U87-GSCs and primary-GSCs when treated by carmustine combined with cilengitide (Supplementary Figure S1). Further cell viability assays indicated that compared to the control group, U87-GSCs and primary-GSCs not grown on FN were markedly and significantly inhibited by carmustine (Figure 4B; *p* < 0.01 for both). However, growing U87-GSCs on 5 or 10 μg/mL FN totally restored cell viability (Figure 4B; *p* < 0.05 and *p* < 0.01, respectively). Only primary-GSCs grown on 10 μg/mL FN showed restored cell viability (Figure 4B; *p* < 0.01). Compared to cells grown on 10 μg/mL FN, cilengitide significantly decreased the cell viability of both U87-GSCs and primary-GSCs (Figure 4B; *p* < 0.05 and *p* < 0.01, respectively). Apoptosis determined by flow cytometry using annexin V–FITC^+^/PI^−^ showed that growing U87-GSCs on 5 or 10 μg/mL FN significantly decreased the apoptosis induced by carmustine (Figure 4C; *p* < 0.01 and *p* < 0.001, respectively). We also investigated cleaved poly (ADP-ribose) polymerase (PARP) expression that is required for apoptosis-inducing factor translocation from mitochondria to the nucleus and that is cleaved at the onset of apoptosis by caspase 3. Compared to U87-GSCs grown without FN, higher levels of cleaved PARP expression were present in U87-GSCs grown without FN or grown on 1 μg/mL FN and treated with carmustine (Figures 4D,E; *p* < 0.01 and *p* < 0.05, respectively). In contrast, cilengitide reversed the anti-apoptotic effect of FN in the presence of carmustine to a slight degree at a concentration of 100 μM (Figures 4D,E). For primary-GSCs, cilengitide alone induced higher expression of cleaved PARP (*p* < 0.05). In accordance with U87-GSCs, higher levels of cleaved PARP expression were present in primary-GSCs grown without FN, or grown on 1 μg/mL FN and treated with carmustine (Figure 4D; *p* < 0.05 for both). In comparison to U87-GSCs, when primary-GSCs were treated with carmustine and cilengitide, they showed significantly higher expression of cleaved PARP (Figures 4D,E; *p* < 0.01). As a tumor suppressor gene, the intrinsic ability of p53 was to mediate apoptotic cell death and to cause cell cycle arrest. Its activity was determined by luciferase luminescence assays. As shown in Figure 4F, the p53 activity of U87-GSCs was elevated slightly without carmustine treatment in cells that grew in the absence of FN. However, p53 activity was markedly increased by carmustine to a peak value on day 1, but declined over the following 2 days (Figure 4F). Compared to U87-GSCs grown in the absence of FN but with carmustine treatment, the p53 activity of U87-GCSs was suppressed markedly when grown on 5 or 10 μg/mL FN, respectively (Figure 4F; *p* < 0.01 and *p* < 0.001 for 5 and 10 μg/mL FN, respectively, vs. 0 μg/mL FN). Restoration of p53 activity was observed in U87-GSCs grown on 10 μg/mL FN and treated with cilengitide (Figure 4F; *p* < 0.01 vs. 10 μg/mL FN).

### Fibronectin Increased Expression of P-Glycoprotein

**Figure 5:**
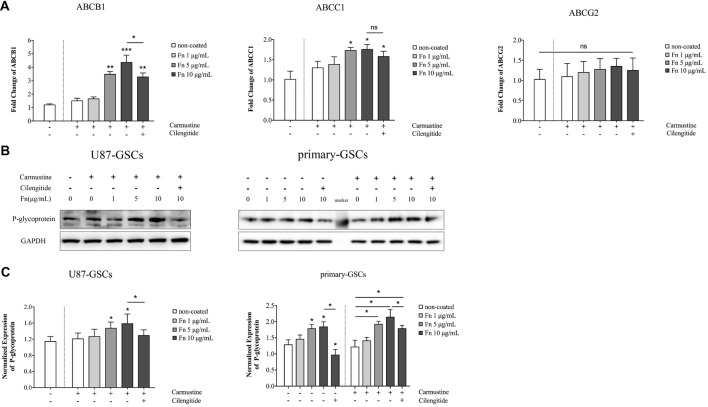
FN increased the expression of P-glycoprotein.** (A)** Quantitative PCR indicated that compared to untreated U87-GSCs, carmustine treatment for 72 h slightly upregulated the gene expression of *ABCB1*, *ABCC1* and *ABCG2* in U87-GSCs, but this was not significant. *ABCB1* was elevated for U87-GSCs grown on 5 or 10 μg/mL FN, which was reversed by cilengitide. The same upregulation of *ABCC1* was observed in U87-GSCs grown on 5 or 10 μg/mL FN, but cilengitide did not reverse this upregulation. A significant difference between each treatment group for *ABCG2* was not noted. **(B,C)** Western blots of P-glycoprotein (*ABCB1*) revealed that growth of U87-GSCs and primary-GSCs on 5 or 10 μg/mL FN led to even higher P-glycoprotein expression compared to U87-GSCs or primary-GSCs grown in the absence of FN. Cilengitide reversed the upregulation of P-glycoprotein. *p < 0.05, **p < 0.01, ***p < 0.001, n.s. not significant.

As proteins embedded within cellular membranes, ABC transporters use energy from the hydrolysis of ATP to move substrates across membranes (Glavinas et al., 2004). Various transporter subtypes are involved in multidrug resistance, including: ABCB1 (also known as multidrug resistance protein 1 or P-glycoprotein), ABCC1 (also known as multidrug resistance-associated protein 1), and ABCG2 (also designated as CDw338; Choudhuri and Klaassen, 2006). To identify whether ABC transporter families were involved in the chemoresistant modulation induced by FN, qPCR was performed to quantify the gene changes mentioned above. Compared to the untreated group, carmustine treatment for 72 h slightly upregulated the gene expression of *ABCB1*, *ABCC1* and *ABCG2*, but this was not significant (Figure 5A). There was a marked elevation of *ABCB1* in U87-GSCs grown on 5 or 10 μg/mL FN (Figure 5A; *p* < 0.01, *p* < 0.001, respectively). Cilengitide significantly reversed this upregulation (Figure 5A; *p* < 0.05). The same upregulation of *ABCC1* was observed in U87-GSCs grown on 5 or 10 μg/mL FN (Figure 5A; *p* < 0.05 for both), but cilengitide did not reverse this upregulation. A significant difference between each treatment group for *ABCG2* was not observed (Figure 5A). Western blots of P-glycoprotein (*ABCB1*) showed that growing U87-GSCs and primary-GSCs on 5 or 10 μg/mL FN led to even higher P-glycoprotein expression compared to the cell lines grown in the absence of FN (Figures 5B,C; *p* < 0.05 for all). Cilengitide reversed the upregulation of P-glycoprotein (Figures 5B,C; *p* < 0.05 for both).

### Fibronectin Activated the FAK/Paxillin/AKT Signaling Pathway

**Figure 6:**
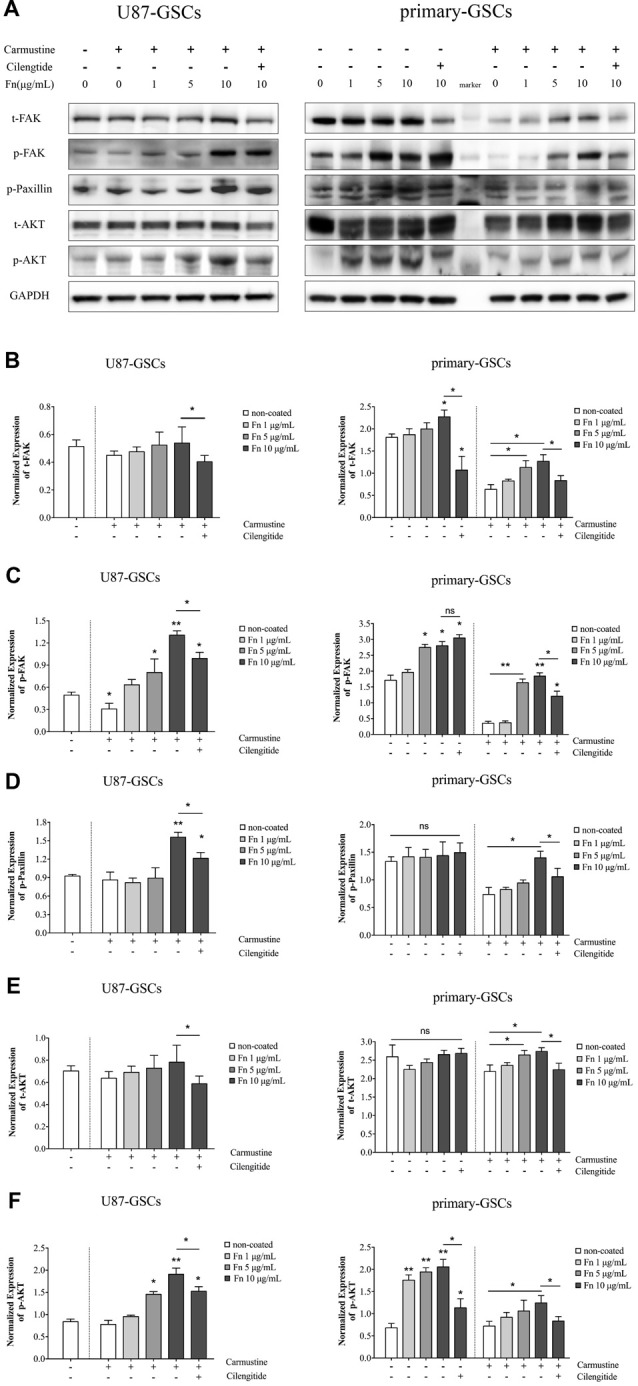
FN activated the focal adhesion kinase (FAK)/paxillin/AKT signaling pathway.** (A–F)** Western blotting indicated that for U87-GSCs, total focal adhesion kinase FAK and AKT expression were not affected by carmustine without cilengitide treatment, whereas cilengitide induced markedly lower FAK and AKT expression than that in the untreated 10 μg/mL FN group. Compared to untreated control, only p-FAK was markedly inhibited by carmustine in U87-GSCs grown in the absence of FN, whereas p-paxillin and p-AKT were not suppressed in U87-GSCs grown in the absence of FN, or U87-GSCs grown on 1 μg/mL FN. In contrast, U87-GSCs grown on 5 or 10 μg/mL FN showed an upregulation of p-FAK, paxillin and p-AKT. Moreover, there was a significant decline of p-FAK, paxillin and p-AKT with cilengitide treatment. For primary-GSCs without carmustine treatment, the expression of p-paxillin and total AKT was not affect by FN. Total FAK was elevated by primary-GSCs grown on 10 μg/mL FN, whereas p-FAK was elevated byprimary-GSCs grown on 5 or 10 μg/mL FN and p-AKT was elevated by primary-GSCs grown on 1, 5 or 10 μg/mL FN. Cilengitide induced markedly lower total FAK and p-AKT expression than that in the untreated 10 μg/mL FN group. The same trend as observed for U87-GSCs occurred when Primary-GSCs were treated with carmustine: primary-GSCs grown on 5 or 10 μg/mL FN showed significantly restored activation of t-/p-FAK and t-AKT compared to carmustine treated cells in the absence of FN, whereas 10 μg/mL FN restored activation of p-paxillin and p-AKT on a large scale. In addition, a marked decline in t-/p-FAK, p-paxillin and t-/p-AKT occurred with cilengitide treatment. *p < 0.05, **p < 0.01, n.s. not significant.

**Figure 7:**
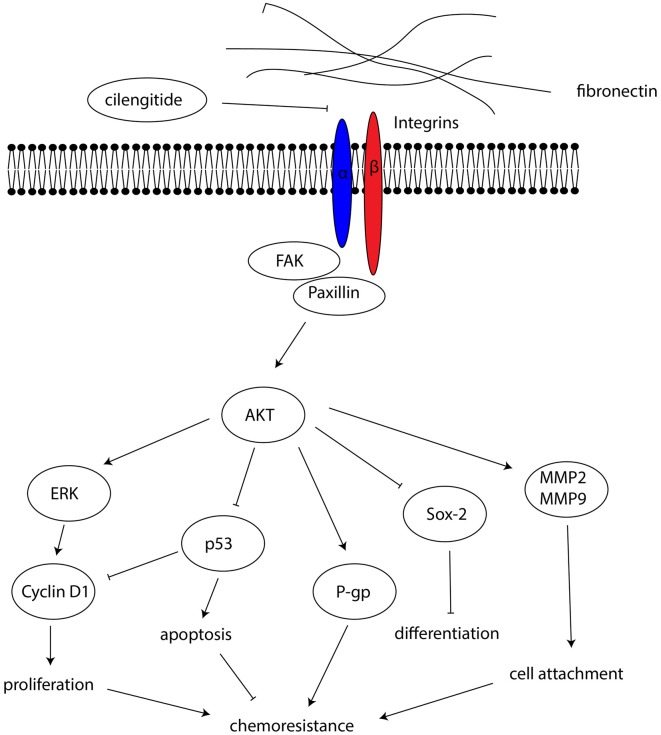
A schematic diagram showing the mechanism by which FN promotes malignancy in GSCs, through effects on cell adhesion, differentiation, proliferation and chemoresistance, via a FAK/paxillin/AKT signaling pathway.

FAK is a signaling molecule that acts as a biosensor that controls cell motility after being activated by various stimuli (Mitra et al., 2005). Activation of FAK and downstream molecules such as paxillin may contribute to cell proliferation, survival and migration through several different signaling pathways (Natarajan et al., 2003; Toutounchian et al., 2017). To investigate the effect on the FAK signaling pathway, FAK, paxillin and AKT proteins were analyzed by western blots after U87-GSCs and primary-GSCs were grown on different concentrations of FN for 72 h. For U87-GSCs grown on 10 μg/mL FN, total expression of FAK and AKT was not affected by carmustine without cilengitide treatment, whereas cilengitide induced significantly lower FAK and AKT expression than that in the untreated 10 μg/mL FN group (Figures 6A,B,E; *p* < 0.05 for both). Compared to the untreated control, only p-FAK was inhibited significantly by carmustine in U87-GSCs grown in the absence of FN (Figure 6C; *p* < 0.05), whereas p-paxillin and p-AKT were not suppressed by carmustine in U87-GSCs grown in the absence of FN or grown on 1 μg/mL FN (Figures 6D,F). In contrast, U87-GSCs grown on 5 or 10 μg/mL FN showed significantly restored activation of p-FAK, p-paxillin and p-AKT (Figures 6A,C,D,F; *p* < 0.05 and *p* < 0.01, respectively, for both treatments except p-paxillin and 5 μg/mL FN). Moreover, a large-scale decline in p-FAK, p-paxillin and p-AKT occurred with cilengitide treatment (Figures 6A,C,D,F; *p* < 0.05 for all). For primary-GSCs without carmustine treatment, the expression of p-paxillin and total AKT was not affected by FN (Figures 6D,E). Total FAK was elevated in primary-GSCs grown on 10 μg/mL FN (Figure 6B; *p* < 0.05), whereas p-FAK was elevated in primary-GSCs grown on 5 or 10 μg/mL FN (*p* < 0.05 for both); p-AKT was elevated in primary-GSCs grown on 1, 5 or 10 μg/mL FN (*p* < 0.05 for both). Cilengitide induced markedly lower total FAK and p-AKT expression in cells grown on 10 μg/mL FN than that in the untreated 10 μg/mL FN group (Figures 6A,B,F; *p* < 0.05 for both). The same trend as observed for U87-GSCs occurred when primary-GSCs were treated with carmustine: Primary-GSCs grown on 5 or 10 μg/mL FN showed significantly restored activation of t-/p-FAK and t-AKT compared to carmustine treated cells in the absence of FN, whereas 10 μg/mL FN restored activation of p-paxillin and p-AKT on a large scale. In addition, a marked decline in t-/p-FAK, p-paxillin and t-/p-AKT occurred with cilengitide treatment (Figures 6A–F; *p* < 0.05 for all). A schematic diagram shows a mechanism of how FN promotes the malignancy of GSCs in cell adhesion, differentiation, proliferation and chemoresistance via a FAK/paxillin/AKT signaling pathway (Figure 7).

## Discussion

In the present study, we demonstrated that FN modulated the biological characteristics of GSCs in many aspects. FN increased the adhesive properties of GSCs as well as their proliferation and their capacity for differentiation in a concentration-dependent manner. We found that FN induced the suppression of p53-mediated apoptosis and that expression of P-glycoprotein was upregulated so that GSCs became chemoresistant to carmustine. In addition, the activation of the integrin FAK/paxillin/AKT signaling pathway was involved in the modulation process. Meanwhile, the integrin inhibitor, cilengitide, reversed the effect on chemoresistance induced by FN.

The establishment of the Cancer Genome Atlas (TCGA) Research Network has led to a classification system based on gene expression patterns that distinguishes four molecular subtypes of GBMs (Cancer Genome Atlas Research Network, 2008). The proneural characteristics of GSCs have been highlighted by past studies (Phillips et al., 2006; Lottaz et al., 2010). Although GSCs present with similar characteristics as neural stem cells, which have multiple potentials to differentiate into all kinds of cells in the central nervous system, glial cells have been the most reported subtype in previous studies (He et al., 2011; Yin et al., 2014). Sox-2 is a key transcriptional factor with a self-renewal potential (Berezovsky et al., 2014), while GFAP and β-tubulin are crucial markers indicating differentiation (Zhang et al., 2014). In the present study, we found increased GFAP and decreased sox-2 at both genetic and protein expression levels, indicating the differentiation of cells in accordance with a previous demonstration. Growing evidence has also revealed that the regulation of the differentiation of GSCs was composed of a complex network that recruited several kinds of regulators and intracellular signaling pathways (Ying et al., 2011; Katsushima and Kondo, 2014). Here, we hypothesize that interaction between FN and its ECM receptor integrin induced the activation of the FAK/AKT/ERK signaling pathway, followed by the suppression of sox-2. However, further investigation is required to support this proposal.

In addition to soluble mitogens, cell proliferation is also regulated by cells adhering via transmembrane receptors such as integrins to ECM molecules such as FN (Danen and Yamada, 2001). However, cell proliferation is regulated by cell adhesion and mitogens only when cells are in the G1 phase (Jakel et al., 2012). D-type cyclins have to be expressed for G1 progression (Vanarsdale et al., 2015). In turn, cyclin D1 depends on the interaction between integrin-mediated cell adhesion and mitogens (Chen et al., 2012). This suggest that the increased expression of cyclin D1 we observed may be responsible for integrin-mediated G1 progression. G1 progression is dependent on cyclin D1 in two ways: First, the initial phosphorylation of Rb is induced by cyclin D–cdk4/6 (Narasimha et al., 2014) that leads to a de-repressed *cyclin E* gene (Kolupaeva and Basilico, 2012). Second, the localization of p21Cip/Waf and p27Kip1 CDK inhibitors (CKI) changes from cyclin E–cdk2 to cyclin D–cdk4/6. In turn, the cyclin E–cdk2 holoenzyme phosphorylates Rb to de-repress the *cyclin* *A* gene (Harbour and Dean, 2000; Aggarwal et al., 2007).

Mammalian cells that undergo DNA damage in response to cell stressors such as chemotherapy or ionizing radiation have an active p53 tumor suppressor that acts to protect the genome (Levine, 1997). In turn, this raises cellular levels of p53 protein and activities (Kastan et al., 1991). Hence, this highlights how p53 regulates the expression of a wide variety of genes in response to DNA damage by acting as a TF during apoptosis, cell cycle arrest, or DNA repair (Fischer, 2017). We found a luciferase luminescence assay to be a sensitive and convenient way to quantify p53 activity (Bellis et al., 2011). The activity of p53 was increased by carmustine at a concentration of 200 μM, but decreased as the FN concentration was gradually elevated, indicating the DNA damage induced by carmustine may be reversed in the presence of FN. We hypothesize that the FN incorporated within integrin receptors leads to the activation of AKT, the downstream molecule regulated by FAK/paxillin pathway. As the key regulator of p53 stabilization and activity (Kubbutat et al., 1997), the murine double minute-2 (mdm2) was enhanced by AKT that is able to promote p53 degradation (Abraham and O’Neill, 2014). Apoptosis mediated by p53 decreased via a mitochondrial or death receptor-induced apoptotic pathway (Vogelstein et al., 2000). However, G_1_ arrest is downregulated due to the decline of p53-dependent p21^Cip/Waf^, which is a CKI (El-Deiry, 1998). The final result is that a decrease of p53 activity induced by FN allowed GSCs to survive.

Chemoresistance induced by the elevation of ABC transporter families has been well demonstrated by others (Martin et al., 2009; Haar et al., 2012). In contrast to a previous notion that ABCG2 may be the key subtype in GSCs (Gong et al., 2014), we found ABCB1, also known as P-glycoprotein, to be the most functional subtype. CD133 and DNA-PK upregulate P-glycoprotein via activation of the AKT–NFκB pathway in multidrug-resistant glioblastoma cells *in* *vitro* (Xi et al., 2016), which is in accordance with our findings.

Acting as cell surface transmembrane molecules, integrins are made up of an α and β subunit. In total, these consist of combinations of 18 α subunits, 10 β subunits and 24 different heterodimeric integrins (Cox et al., 2010). FN binds to integrins and triggers intracellular signaling through the activation of FAK and its downstream molecules (Digiacomo et al., 2017). In the present study, we found that attachment to FN led to the activation of the FAK/paxillin/AKT signaling pathway, and this was associated with the proliferation and differentiation of GSCs. Integrins in malignant gliomas have been associated with many cellular functions, including angiogenesis, invasion, migration and adhesion (Abdollahi et al., 2005). The regulation of integrins is typified by both by “outside-in” and “inside-out” signaling, the latter causing the extracellular portion of the integrin’s subunits to change its conformation, and either become switched on or de-activated. In this manner, the activation of integrins is dependent on a complex crosstalk network causing bidirectional signaling (Tabatabai et al., 2011). Our data showing differences in the degree of the detaching effect of cilengitide on U87-GSCs and primary GSCs may be due to the differing enrichment of integrin αV receptors on the cell surface, which also led to a varied degree of restoration of the activation of FAK/Paxillin/AKT signaling when cells grew on FN. Interestingly, Worthington et al. (2011) discovered that a latent complex composed of TGF-β and its corresponding receptor is activated by αvβ8. In this manner, TGF-β function may be regulated and this may explain how the effects of TGF-β only occur in microenvironments that can activate the latent complex (Worthington et al., 2011).

Here, we demonstrate that FN is an important ECM component that can modulate the biological behavior of U87-GSCs and primary-GSCs in terms of cell adhesion, proliferation, and differentiation in a concentration-dependent manner. Particularly, attachment to FN led to chemoresistance to carmustine. The integrin inhibitor, cilengitide, reversed the chemoresistant effect. The activation of FAK/paxillin/AKT was involved in the regulatory effects of FN on U87-GSCs and primary-GSCs. Further investigation targeting the ECM is required to understand the crosstalk between tumor cells and their microenvironment, suggesting a new direction for the development of novel chemotherapies for glioblastoma.

## Author Contributions

YL and YX conceived and designed this study and revised the article critically. QY, JL and ZX performed the main experiments. JL and ZX helped with the lentiviral construction assay. ZL helped with the bioluminescence assay. QY drafted the manuscript and performed the literature review. All authors had final approval of the submitted versions.

## Conflict of Interest Statement

The authors declare that the research was conducted in the absence of any commercial or financial relationships that could be construed as a potential conflict of interest.

## Supplementary Material

FIGURE S1 After U87 glioma stem-like cells (U87-GSCs) and primary-GSCs were cultured on pre-coated fibronectin (FN) and treated with 200 μM carmustine for 72 h, increasing numbers of cells survived as the concentration of fibronectin increased. Moreover, increased cell death was observed for both U87-GSCs and primary-GSCs when treated by carmustine combined with 100 μM cilengitide.

FIGURE S2 After U87-GSCs were cultured on pre-coated FN for 72 h, immunofluorescence staining revealed FN at 5 or 10 μg/mL induced increased expression of Ki-67, indicating FN promoted cell proliferation. Images were taken at the same exposure settings. Cells grown without FN and stained without primary antibody were used as a negative control. ^*^*p* < 0.05.
