# Supplementary figures and images for: Fibronectin Promotes the Malignancy of Glioma Stem-Like Cells Via Modulation of Cell Adhesion, Differentiation, Proliferation and Chemoresistance
Source: Front Mol Neurosci. 2018 Apr 13;11:130. doi: 10.3389/fnmol.2018.00130 (PMC5908975; doi:10.3389/fnmol.2018.00130)

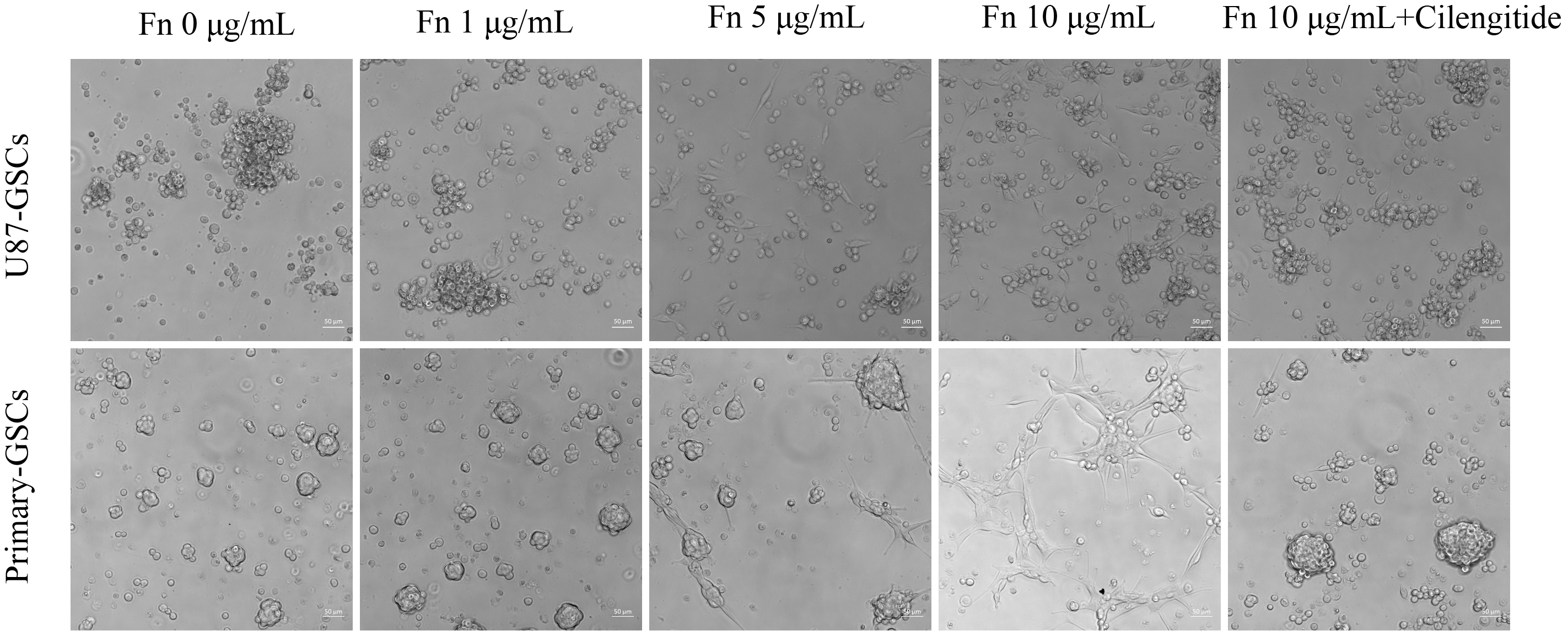

Supplement: FIGURE S1 — After U87 glioma stem-like cells (U87-GSCs) and primary-GSCs were cultured on pre-coated fibronectin (FN) and treated with 200 μM carmustine for 72 h, increasing numbers of cells survived as the concentration of fibronectin increased. Moreover, increased cell death was observed for both U87-GSCs and primary-GSCs when treated by carmustine combined with 100 μM cilengitide. [file Image_1.JPEG]

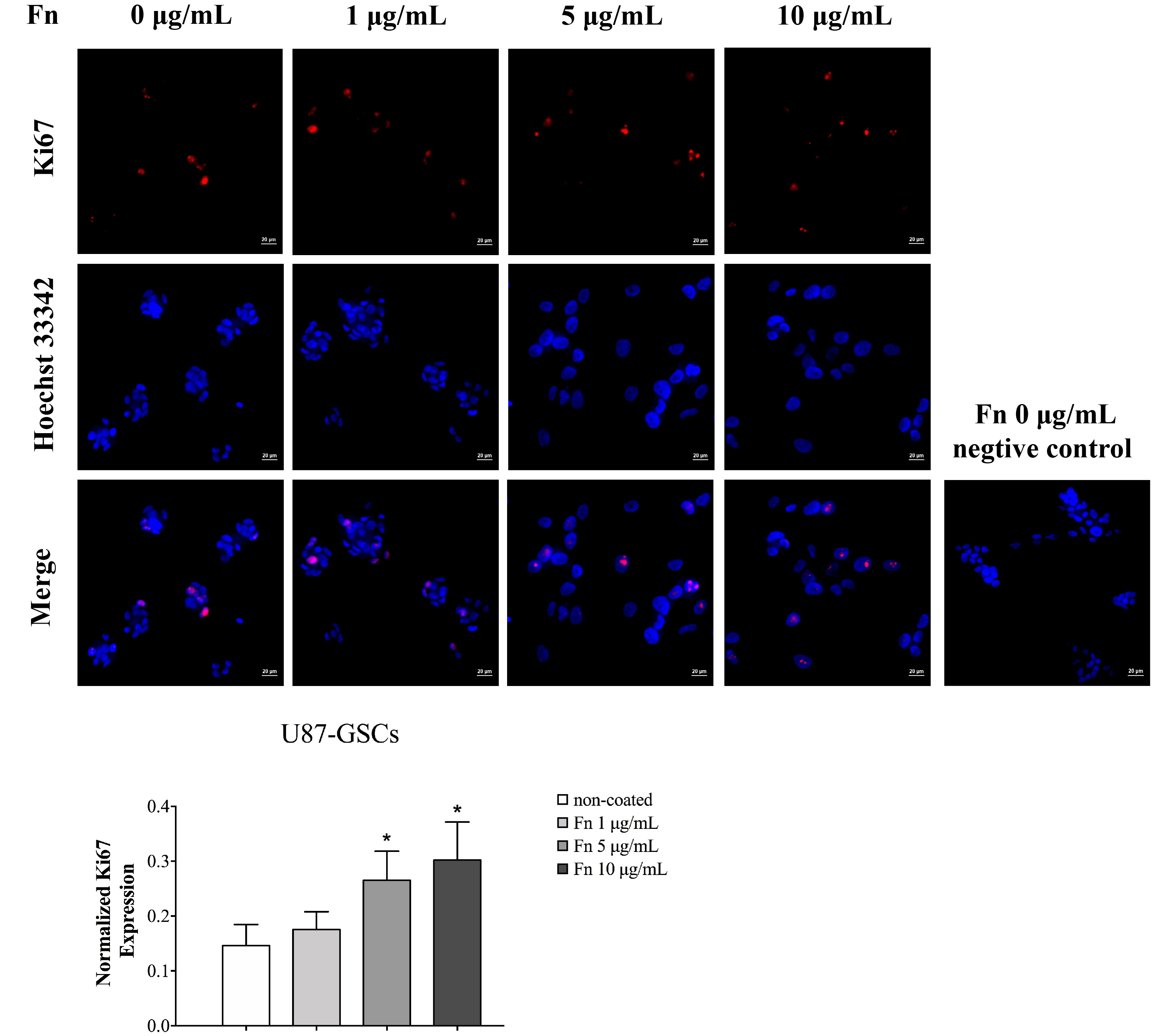

Supplement: FIGURE S2 — After U87-GSCs were cultured on pre-coated FN for 72 h, immunofluorescence staining revealed FN at 5 or 10 μg/mL induced increased expression of Ki-67, indicating FN promoted cell proliferation. Images were taken at the same exposure settings. Cells grown without FN and stained without primary antibody were used as a negative control. *p < 0.05. [file Image_2.JPEG]
